# Plant Resources Utilization among Different Ethnic Groups of Ladakh in Trans-Himalayan Region

**DOI:** 10.3390/biology10090827

**Published:** 2021-08-26

**Authors:** Shiekh Marifatul Haq, Umer Yaqoob, Eduardo Soares Calixto, Inayat Ur Rahman, Abeer Hashem, Elsayed Fathi Abd_Allah, Maha Abdullah Alakeel, Abdulaziz A. Alqarawi, Mohnad Abdalla, Musheerul Hassan, Rainer W. Bussmann, Arshad Mehmood Abbasi, Sami Ur Rahman, Farhana Ijaz

**Affiliations:** 1 Department of Botany, University of Kashmir Srinagar, Srinagar 190006, India; marifat.edu.17@gmail.com (S.M.H.); umer.scholar@kashmiruniversity.net (U.Y.); 2 Wildlife Crime Control Division, Wildlife Trust of India, Noida 201301, India; 3 Institute of Biology, University of São Paulo, São Paulo 05315-970, Brazil; calixtos.edu@gmail.com; 4 Department of Botany, Hazara University, Mansehra 21300, Pakistan; fbotany@yahoo.com; 5 Botany and Microbiology Department, College of Science, King Saud University, Riyadh 11451, Saudi Arabia; habeer@ksu.edu.sa (A.H.); 441203162@student.ksu.edu.sa (M.A.A.); 6 Department of Plant Production, College of Food and Agriculture Science, King Saud University, Riyadh 11451, Saudi Arabia; eabdallah@ksu.edu.sa (E.F.A.); alqarwy@ksu.edu.sa (A.A.A.); 7 Key Laboratory of Chemical Biology (Ministry of Education), Department of Pharmaceutics, School of Pharmaceutical Sciences, Cheeloo College of Medicine, Shandong University, 44 Cultural West Road, Jinan 250012, China; mohnadabdalla200@gmail.com; 8 Clybay Research Private Limited, Bangalore 560114, India; musheer@clybay.com; 9 Department of Ethnobotany, Institute of Botany, Ilia State University, 1 Botanical Street, Tbilisi 0105, Georgia; rainer.bussmann@iliauni.edu.ge; 10 Department of Environmental Sciences, Abbottabad Campus, COMSATS University Islamabad, Islamabad 22060, Pakistan; amabbasi@cuiatd.edu.pk; 11 Nawaz Sharif Kidney Teaching Hospital and Postgraduate Institute Manglawar, Swat 19200, Pakistan; drsamiurrahman.swat@gmail.com

**Keywords:** biodiversity, ethnobotanical uses, plant resource, Ladakh, chord diagram, trans-Himalayas

## Abstract

**Simple Summary:**

Indigenous communities are a large resource of increasingly endangered, traditionally used medicinal plants and the associated ecological knowledge, which needs to be documented quickly as the base to establish sustainable livelihoods and healthcare systems. Through the interaction of indigenous knowledge, biodiversity, and the surrounding environment, these communities have developed their livelihoods over time. In this study, we tried to obtain an in-depth understanding of ethnomedicinal, cultural, and ritual perspectives on plant diversity in the Ladakh region and evaluated how the wild flora of Ladakh could improve local livelihoods and alleviate poverty. By surveying and applying open- and close-ended semi-structured interviews and group discussions in three communities, we documented 105 ethnobotanically important plants belonging to 39 families. The Balti and Brokpa ethnic groups showed greater similarity, whereas the least overlap in plant use was observed between Beda and Brokpa. Plants common to all cultures were mostly used for medicinal applications, while some were also used for religious purposes in the two major religions (Islam and Buddhism). A total of 37 species were shared by all cultures (Balti, Brokpa, and Beda). The cluster analysis elucidated three major clusters of different ethnobotanical usage. The first cluster included food and medicinal plants, the second included clusters of dye and flavor plants, and the third included plants used for fragrance, oil, fuel wood, and fodder. Plant parts were collected based on their availability in distinct pheno-phases according to the Tibetan traditional calendar. This study’s findings revealed that plants provide tangible economic benefits to indigenous communities, in addition to aiding in the treatment of various ailments. Sustainable use and management of wild resources can help improve livelihoods and food security and alleviate poverty.

**Abstract:**

The nomadic pastoral indigenous communities of the Ladakhi people share roots with Tibetan culture in terms of food, clothing, religion, festivals, and habits, and rely widely on plant resources for survival and livelihood. This survey was conducted during 2019–2021 to document the indigenous knowledge about plant resources of the Balti, Beda, and Brokpa communities of the Ladakh region, trans-Himalayas. Open- and close-ended semi-structured interviews (N = 184) and group discussions (N = 17) were used to collect the data. Quantitative data was further analyzed using various statistical tools. A total of 105 plant species belonging to 82 genera and 39 families were used as medicine, fuel wood, fragrance, oil, food, flavor, fodder, decoration, and dye. Among these, medicinal use was most prevalent, with 70% of use reports, followed by fodder and fuel wood. Leaves (27%) were the most preferred plant part used, followed by roots and flowers. The principal component analysis revealed five clusters of ethnobotanical usage, i.e., food, medicine, fuel wood, fodder, and fragrance, oil, dye, and flavor. The maximum number of plant species used was reported by the Brokpa, while the Beda reported the minimum number of plant species uses. *Delphinium brunonianum*, *Waldheimia tomentosa*, and *Juniperus indica* played a significant role in the cultural and religious ritual aspects, whereas *Allium przewalskianum*, *Waldheimia tomentosa*, *Juniperus indica*, and *Hippophae rhamnoides* were commonly used as a livelihood source among Ladakhi communities. The local people collected most plants (65%) for self-consumption, while the rest (35%) were sold in markets as a source of income. The sustainable utilization and management of plant resources by local people is a strategy to boost livelihoods and food security and alleviate poverty.

## 1. Introduction

Humanity has always been dependent on ecosystem services [1]. Plant biodiversity throughout the globe has been providing these ecosystem services in terms of both economy and culture [2], including food and fodder for humans and their livestock, timber, firewood, and herbal remedies for treating various ailments [3]. Many plant resources have cultural importance, e.g., in education, in religion, as totems, for aesthetics, etc., and are important for socio-economic and industrial activities [4,5].

Indigenous communities have developed broad ecological knowledge and are, often, still dependent on wild plants for food, fodder, medicines, and other purposes. The focus on traditional plant foraging is especially vital in remote tribal areas in comprehending its role in the sustainability of food systems and for the promotion and discovery of novel local gastronomies [6]. Ethnomedical practices have resulted in the development of traditional medicinal systems such as Unani, Siddha, Sowa-Rigpa, and Ayurveda and are also part of many even allopathic drugs [7]. The population of developing countries is especially reliant on these traditional plant-based medicines due to the lack of modern health facilities [8]. Ethnobiological field studies have widely shown that indigenous communities represent a significant reservoir of disappearing folk plants and ecological knowledge, which needs to be immediately documented to develop sustainable food and healthcare systems [9]. Wild plants play an imperative role in the livelihood of tribal people [10]. Over the years, traditional knowledge has resulted in the development of systems providing livelihood to the indigenous communities. These sustainable livelihoods are developed over the years through the interaction of indigenous knowledge, biodiversity, and the surrounding environment [11]. Traditional knowledge has also been proved to be effective in the conservation of biological resources, which are important stakeholders in conserving biodiversity. This complex relationship is difficult to manage by outside experts. Thus, to conserve the diversity of different plants used by indigenous communities, it is imperative to involve indigenous communities in conserving them as they know how the different interaction factors work with each other [11].

Ladakh has a rich history of medicinal and wild food plants, but limited studies on the utilization of wild plant species that could help in identifying novel and potential sources of medicines, food, and other plant products have been carried out to document the associated traditional knowledge. The Sowa-Rigpa herbal medicine system is considered the oldest codified healthcare system known to humanity [12]. The conservation of the records of this valuable knowledge is as imperative as medicinal plant conservation [13]. Our study quantified the indigenous ethnomedicinal uses of plants and assessed the distribution, composition, conservation, and trade of medicinal plants of Ladakh. The main objectives of the fieldwork were (1) to gain an in-depth understanding of ethnomedicinal, cultural, and ritual perspectives of plant diversity in the Ladakh region and (2) to evaluate how the wild flora of Ladakh could boost up livelihood and food security and support in poverty alleviation. Various studies have reported medicinal and other ethno uses of plants from Ladakh [14,15]; however, the local population in our study area (Balti, Beda, and Brokpa) has never participated in an ethnobotanical study.

## 2. Materials and Methods

### 2.1. Study Area

**Figure 1:**
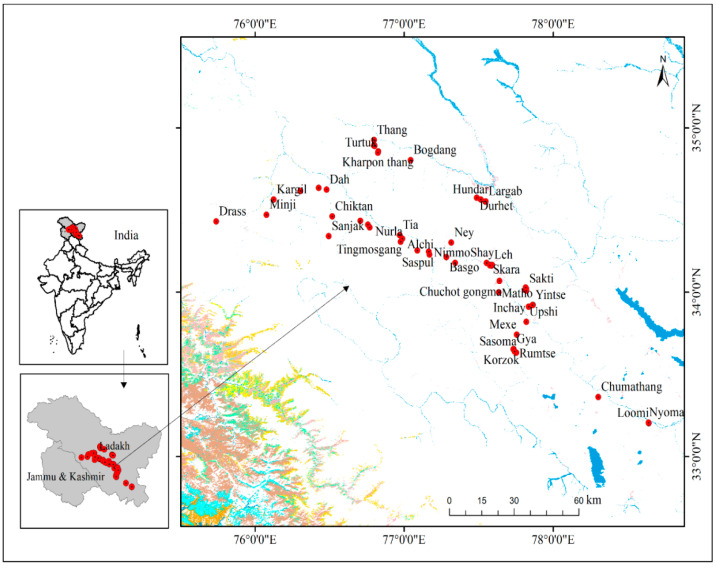
Map of the study area showing the sampling sites (*n* = 54) in the Ladakh trans-Himalayan region, India. A global positioning system (GPS Garmin map76cs) was used to record the altitude as well as geo-coordinates of the sampled sites.

The Ladakh region in the trans-Himalayas of the Indian Himalayan Region (IHR) is one of the highest plateaus in the world. The study area is part of the previously northern Jammu and Kashmir state and is located at 34°12′34.25″ N and 77°36′54.40″ E (Figure 1). Ladakh covers less than half of the landmass of the state and is surrounded by Tibet (China) to the north and northeast, Gilgit-Baltistan (Pakistan) to the northwest, and Himachal Pradesh to the west. The total area of Ladakh is 59,146 km^2^, which as per the 2010 census had a population of 290,492, with a population density of 4.91 people/km^2^, representing the lowest density in the country. Administratively, Ladakh has two districts: Kargil, with 14,036 km^2^ area and about 143,388 inhabitants, and Leh district (45,110 km^2^, 147,104 inhabitants) [16]. The largest town in Ladakh is Leh, located at an altitude of 3000 m, followed by Kargil at 2676 m, each of which headquarters a district. The area is cold and dry, with an annual rainfall of less than 120 mm. In winter, the entire area is covered with heavy snow. Most of the settlements are in the river valleys. The flora of Ladakh is a rich source of aromatic and medicinal plants and may be grouped into three broad classes—arid vegetation, alpine mesophytes, and oasitic or riparian vegetation [17].

Leh comprises the Indus, Shyok, and Nubra river valleys, while Kargil includes the Suru, Dras, and Zanskar river valleys. The Nubra, Shyok and Indus River valleys are the most populated areas of the district Leh. The main ethnic groups include Balti, Beda, and Brokpa, all living across both divisions. The Balti tribe is originally of Tibetan descent and mainly found in the Kargil region, although a small population also lives in Leh. The spoken language is known as Balti. The Beda are a unique tribe famous for their musicianship (traditional occupation) and scattered in Ladakh. They predominantly follow the Muslim faith. The Brokpa (people living in mountains) are a small community of Dard people found in Leh and Kargil. Those living in Leh are Buddhists and the inhabitants of Kargil are Muslims. A small number of people have embraced Hinduism. Their language is known as Brokstat.

### 2.2. Data Collection

The present study was carried out over the period 2019–2021 in the Balti, Beda, and Brokpa ethnic groups. Information was gathered through open- and close-ended semi-structured interviews (N = 184) and group discussions (N = 17) following [16]. Participants (N = 269) included 77.32% men and 22.67% women, of which 7.80% were Amchis (traditional doctors), herders (8.92%), hunters (5.94%), shopkeepers (4.83%), farmers (30.48%), daily wage laborers (7.80%), hotel owners (5.57%), museum owners (0.74%), housewives (19.33%), and government employees (8.55%). Interviews and discussions focused on the ethnobotanical use of local plant resources as medicine, food, fodder, fuel wood, fragrance, dye, decoration, flavor, and oil, including information on harvesting time and season. The ethnicity of the participants and the language information given here are not disclosed, based on mutual agreement as stipulated under the Nagoya Protocol. The code of ethics of the International Society of Ethnobiology was followed [18]. Additionally, one person from each indigenous community, who was well familiar with the traditions and norms of the community, was taken as a guide during all the field surveys. Information was gathered about key plant species used for cultural, religious, and ritual beliefs, and compared to [13,19].

Plant specimens were collected from different sites during the field survey and were properly coded/tagged. Specimens were identified with the help of taxonomists at the CBT Lab, University of Kashmir, Srinagar (J&K), by comparing with herbarium specimens at the KASH herbarium and local floras [20]. The nomenclature and botanical families of all the specimens were further authenticated using www.plantsoftheworldonline.org/ (accessed on 28 November 2020).

### 2.3. Data Analysis

Analyses of the ethnobotanical uses of plant species were carried out using cluster analysis. We used absence/presence data to show the distribution of the species, clustering species with similar ethnobotanical uses using PAST software ver. 3.14. Sørensen’s (Bray–Curtis) distance similarity coefficient, based on presence/absence data, was used to identify significant differences among diverse ethnobotanical uses and plant species [21,22]. Principal component analysis (PCA) was performed to visualize provisioning services and plant parts used, using the package “vegan” [23] in the software R 4.0.0 [24]. To evaluate whether there was a difference in the number of plant parts used, we used a generalized linear model (GLM) with binomial distribution, followed by the likelihood-ratio test. The contribution of different plant parts used was displayed in chord diagrams using the circlize package [25] in R software 3.6.1 [23]. The Venn diagram was created using Bioinformatics & Evolutionary Genomics software (http://bioinformatics.psb.ugent.be/cgi-bin/liste/Venn/calculate_venn.htpl (accessed on 21 January 2021)).

## 3. Results and Discussion

The results of the study revealed that the local population of the study area is still a rich source of herbal medicines and traditional knowledge. Furthermore, the current work evidently indicates the close connection between the local population and provisioning ecosystem services of plants.

### 3.1. Demographic Details of Respondents and Vegetation Composition

In the present study, the respondents represented a diverse array of professional groups, including daily wage laborers, farmers, government employees, herders, hotel owners, hunters, homemakers, museum owners, shopkeepers, and traditional doctors (Amchis), across three ethnic groups, i.e., Beda (N = 63), Balti (N = 91), and Brokpa (N = 115; Table 1). Among the 269 respondents, 77.3% were men, and the remaining 22.7% were women. A possible reason for the smaller number of female informants is that they are confined to their homes due to cultural restrictions [26,27]. Most of the informants (48%) were 46–65 years old, followed by 66–88 (40%) and 25–45 (12%). More than half of the respondents were without formal education (65%; Table 1). We noticed that older people hold more traditional knowledge than younger people in this area, a fact also reported in earlier studies [28]. As in other parts of the Himalayan region, ethnic knowledge about the uses of different therapeutic plants was decreasing in the younger people of the study area, which may be ascribed to little interest shown by the younger generation in inheriting and using ethnomedical practices [29]. In addition, the illiterate population was found to have more ethnomedical information, which may be ascribed to the fact that educated participants are expected to be exposed to the developed world and mostly rely on modern medicines instead of traditional ones [30]. During the survey, it was noticed that the population in rural areas also had more knowledge of natural resources compared to urban populations.

**Figure 2:**
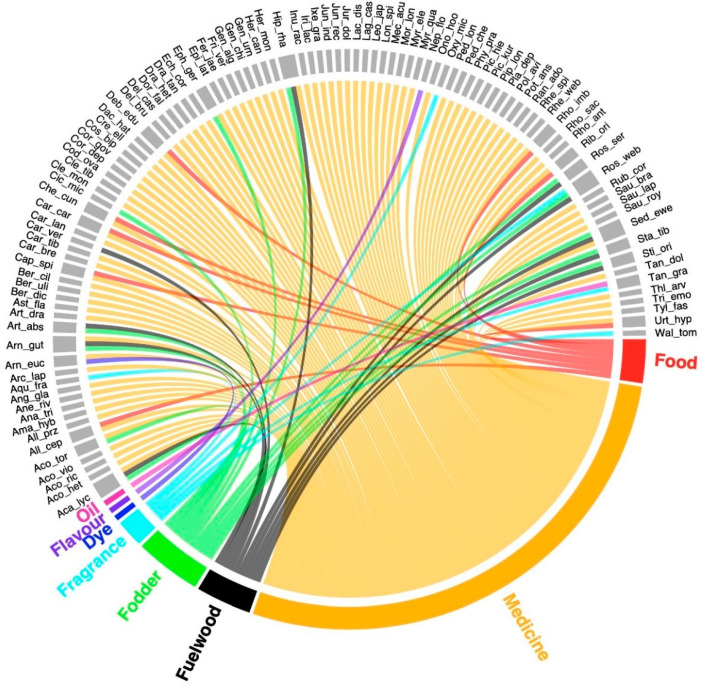
Plant species distribution (105 species) according to plant usage in the Ladakh trans-Himalayan region, India.

In this study, we documented 105 ethnobotanically important plants belonging to 82 genera and 39 families (Table 2). The respective uses, i.e., medicine, fuel wood, fragrance, oil, food, flavor fodder, decoration, and dye, are presented in Figure 2. The number of plant species recorded in the study area was close to those documented by earlier ethnobotanical studies in other parts of the Himalayan region. Bhattarai et al. [26] and Ambu et al. [31] reported 121 and 116 species from the trans-Himalayan region of Nepal. Awan et al. [32], Mulk et al. [33], and Ajaib et al. [34] reported a total of 102, 101, and 100 plant species, respectively, from the Western Himalayas of Pakistan. Similar results were reported by Rana and Rawat [35], Kayani et al. [36], and Haider and Qaiser [37] in the Himalayan region. During the field work, it was noted that medicinal plant richness decreased with altitude, while the percentage of use reports of medicinal plants also gradually increased with altitude. This may be a result of the preference given by the local population to medicinal plants from higher-altitude areas. Lone et al. [38] also reported similar results from the Bandipora district of Jammu and Kashmir.

The distribution of the collected plant species in the 39 families was uneven. About half of the collected plant species belonged to just six families, i.e., Asteraceae, Ranunculaceae, Fabaceae, Apiaceae, Lamiaceae, and Polygonaceae, while the remaining half belonged to 32 families. Most of the genera (19) were monotypic (Table 2). Because of their wide range of ecological amplitudes, Asteraceae adapt easily in arid and dry habitats [39,40]. Several studies have found Asteraceae to be a dominant family in surrounding areas [28,41], although Kayani et al. [36] reported Ranunculaceae as the most dominant family from the high-altitude areas of Pakistan. Similar findings were reported by Bhattarai et al. [26] from trans-Himalayan Nepal, Ijaz et al. [42] from the Pakistani Himalayas, and Kala [43] from trans-Himalayan India. Kayani et al. [36] and Abbas et al. [44] found Fabaceae and Ranunculaceae to be prominent families from the Pakistani Himalayas. Similarly, Debbarma et al. [45] reported Fabaceae as the dominant family in northeast India. However, Pala et al. [46] reported Lamiaceae as the leading family from the Eastern Himalayas, which is in line with our results. Similar species distribution patterns were observed by other ethnobotanical studies from the Himalayas [47,48]. The large number of therapeutic plants from the families Asteraceae, Apiaceae, Fabaceae, Lamiaceae, Ranunculaceae, and Polygonaceae is possibly due to the abundance and wider distribution of these families in this area [43]. Furthermore, according to various researchers [49,50,51], the members of these families have a high content of useful bioactive compounds.

### 3.2. Preference Analysis

**Figure 3:**
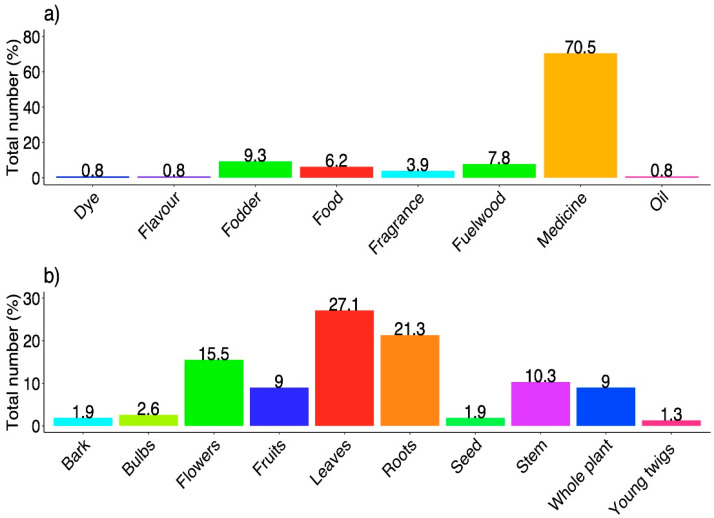
(**a**) Percentage of different ethnobotanical usages; (**b**) percentage of different plant parts used in the Ladakh trans-Himalayan region, India.

We emphasized the numerous ethnobotanical uses of the reported species among the local communities. The results obtained through preference analysis indicated a considerable variance (χ^2^ = 408.56, df = 7, *p* < 0.001) in plant usage between the different communities. Medicinal use was overall the most prevalent, with 70% of use reports, followed by fodder, fuel wood, food, fragrance, dye, flavor, and oil (Figure 3a). This demonstrates that local communities prefer the traditional “Sowa-Rigpa” (ancient Indian medicinal system, which evolved in the entire trans-Himalayan region) healthcare system [52]. This also reflects the demand of the pharmaceutical industry, given the high market value for medicinal species [53,54]. Ijaz et al. [42] reported similar results from Pakistan. Haq et al. [48] also reported maximum usage of plants for medicinal purposes from the Northwestern Himalayas, followed by other ethnobotanical uses. Other studies [54,55,56,57] found similar results.

**Figure 4:**
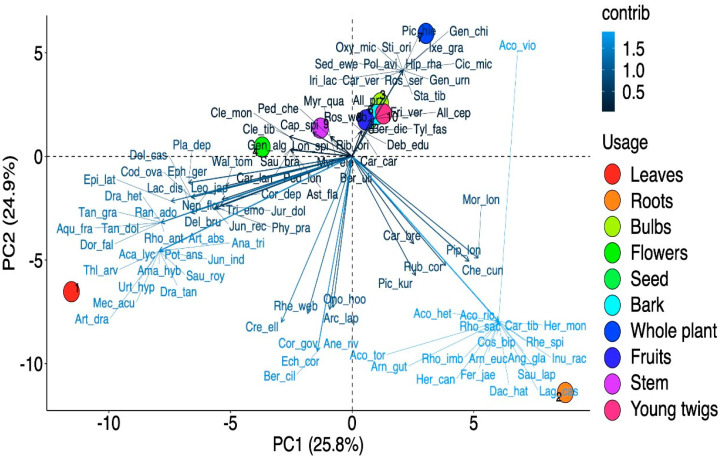
Principal component analysis (PCA) biplot of different part(s) usage in the Ladakh trans-Himalayan region, India.

The indigenous community used different plant parts for various ethnobotanical uses (Figure 3b) with a significant difference (χ^2^ = 100.12, df = 9, *p* < 0.001) between their usage. The results obtained through preference analysis indicated a noteworthy variance, with leaves (27%) the most used, followed by roots, flowers, stem, fruits, whole plant, bulbs, bark, seeds, and young twigs (Figure 3b). The PCA analysis also supported our results and showed ten individual groups centered on the variations in the preference levels of plant parts usage (Figure 4). PC1 and PC2 explained 50.7% of the parts used in the biplot, in which ten clusters of plant part usage based on species presence/absence can be identified: leaves, roots, bulbs, flowers, seeds, bark, whole plant, fruits, stem, and young twigs (Figure 4). Due to the dependence of local people on wild plant resources for daily cuisine, different plant parts are preferred according to their uses. Leaves are the main photosynthetic organs and thus contain lots of metabolites [28,58]. Furthermore, using leaves and aerial parts is regarded as safe as well as sustainable [59]. Roots are also known to contain a good concentration of bioactive compounds [60,61], and local shepherds, Amchis and herbal drug dealers, and other ethno-groups often prefer to use/trade the roots of plants for medicinal purposes [62].

The overharvesting of underground parts or whole plants should be discouraged, especially in the case of threatened species, as this practice causes elimination and dwindling of the plant’s status in the wild [43,63]. Our findings are also supported by Ahmad et al. [64], Sharif et al. [65], Siddique et al. [66], Anwer et al. [67], and Manduzai et al. [68] from the Pakistan Himalayas; and Debbarma et al. [45] and Krupa et al. [69] from India. Asif et al. [28] and Haq et al. [48] from the Kashmiri Himalayas, India; Pala et al. [46] from the Eastern Himalayas; Singh et al. [70] from the Western Himalayas, India; and Tiwari et al. [71] from the Kumaun Himalayas, India. The collection of plant parts is designed depending upon the availability of plant parts in various pheno-phases following the Tibetan traditional calendar [72]. For example, leaves were collected in spring (April and May), flowers and mature leaves in summer (June and August), and, finally, fruits, roots, and seeds in autumn (September and November). The rural inhabitants, herders, Amchis, and elderly people were aware of plant collection timings and selective harvesting of plants for ethnobotanical usage. A similar pattern of plant part collection was reported by Lone et al. [38], Kala [72], Ghimire et al. [73], and Kala [74], from the Himalayas [38,72,73,74].

Wild leafy vegetables such as *Allium przewalskianum*, *Amaranthus spinosus*, *Plantago depressa*, and *Urtica hyperborea* growing close to and in human settlements were especially frequently used. The leaves of *Urtica hyperborea* were commonly used for making soup by the Buddhist inhabitants of Leh. It was also noted that for herbal preparations, plant parts were mostly used in dried form and the reason for this was that the dried plant parts were kept for later seasons, particularly for the winter season [48]. Most of the formulations were prepared and administered at home, like in the results of Lone et al. [38]. The local people in their respective localities were sometimes assisted by other knowledgeable people, when necessary, with no or very low charges. However, it was stated by most of the informants that they kept their knowledge of medicinal plants secret. Furthermore, they revealed that the sharing of traditional knowledge of medicinal plants may take place only with family members, mostly from parents to sons, which is one reason why, in the present study, it was documented that men have more knowledge about medicinal plants than women.

### 3.3. Cross-Cultural and Religious Analysis

**Figure 5:**
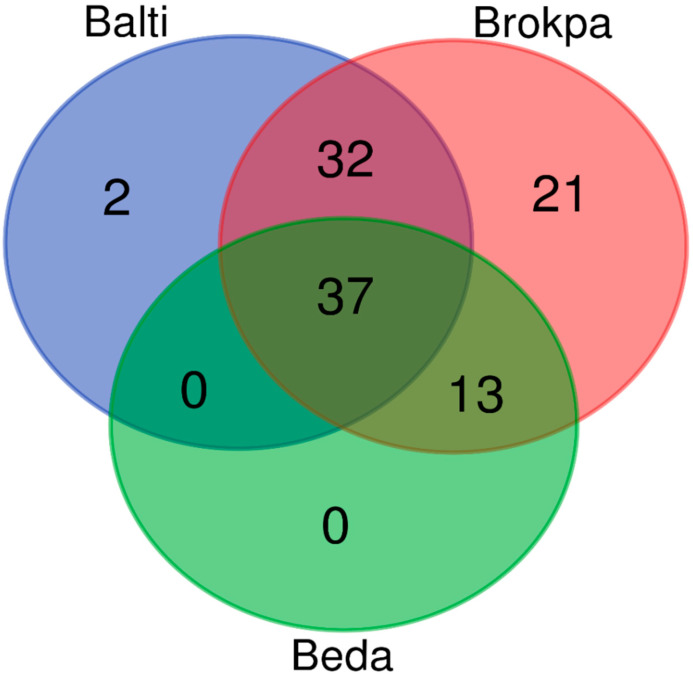
Venn diagram showing the overlap of ethnobotanical usage of plants in different ethnic groups in the Ladakh trans-Himalayan region, India.

The Venn diagram (Figure 5) shows that the maximum number of plant uses was reported by the Brokpa, while the Beda reported a minimum number of plant uses. The Balti and Brokpa ethnic groups showed greater similarity, whereas the least overlap was observed between Beda and Brokpa. A cross-cultural comparison of plant resources showed that 37 plants were commonly used by all ethnic groups. Gairola et al. [75] also reported on the cross-cultural usage of plants from the Himalayas. Plants common among all cultures mainly had medicinal value, although certain plants were common because of their religious uses in the two main religions (Islam and Buddhism). Some species were found in all cultures (Balti, Brokpa, and Beda).

Many of the plants used in the wider region play a significant role in some cultural and religious ceremonies [76,77]. *Delphinium brunonianum* (Ba-ru-ra/Ladar) was used by local healers in dealing with evil spirits. *Waldheimia tomentosa* (Palu) is an aromatic holy plant which was used as incense (locally known as dhoop) in houses and religious places on auspicious religious and cultural days. Palu was also used in the bathing ceremony for deceased persons and in the baby shower of newborns in the Buddhist faith. The dried leaves of *Waldheimia tomentosa* were added to hot water and then used during bathing. Similarly, it was found that *Juniperus indica* (Shukpa) was used as incense by Buddhists in their monasteries and in religious and marriage events, as well as in dealing with nightmares. The dried leaves of this plant were burned in a mud pot to produce smoke, which was kept in front of people who were dealing with bad dreams, especially by Muslims. Plant species such as *Codonopsis ovata* and *Cremanthodium ellisii* were used to fend off evil spirits. The seeds of *Datura stramonium* (Esman) were used by the Balti tribe for dealing with evil spirits. Branches of *Salix pycnostachya* Andersson (Malchang) were used as pillows for corpses in graves (Muslim faith). A similar usage of plant resources for religious and ritual beliefs was reported by Amjad et al. [78] from Pakistan and Sharma et al. [79] from Assam, India.

### 3.4. Classification of Ethnobotanical Usage

**Figure 6:**
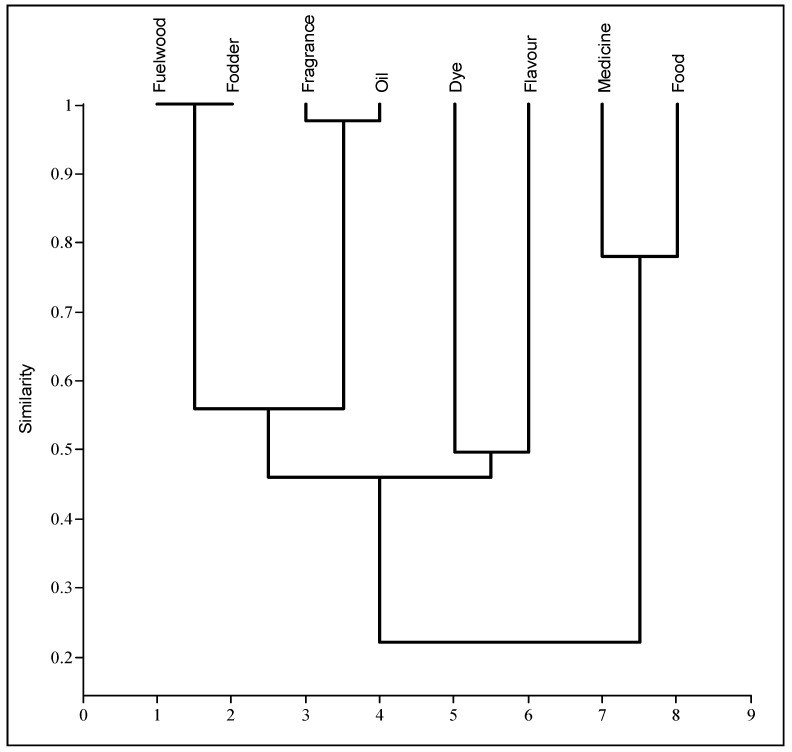
Cluster diagram of the different provisioning services based on plant usage patterns in the Ladakh trans-Himalayan region, India.

**Figure 7:**
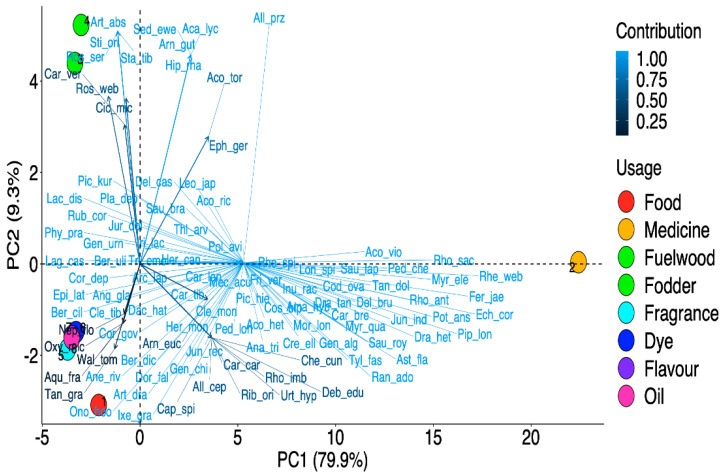
Principal component analysis (PCA) biplot of different provisioning services in the Ladakh trans-Himalayan region, India.

Cluster analysis elucidated three clusters of different ethnobotanical uses based on floristic similarity. The first cluster included food and medicinal plants, the second included dye and flavor plants, and the third included plants used for fragrance, oil, fuel wood, and fodder (Figure 6). Species such as *Amaranthus spinosus*, *Allium humile*, and *Allium przewalskianum*, having both medicinal and food value, were grouped in the first cluster. Plants such as *Artemisia absinthium* and *Oxytropis microphylla*, used as dyes as well as flavoring agents, fell into the second cluster. Plants such as *Caragana versicolor* and *Hippophae rhamnoides*, used as fodder as well as fuel wood and oil sources, formed a separate, third cluster. The principal component analysis (PCA) also supported these results, showing distinct use clusters based on variations in the preference levels (Figure 7). The PCA correlated the most important components with other underlying variables. PC1 and PC2 explained 89.2% of the provisioning services in the biplot, in which five clusters of ethnobotanical usage based on species presence/absence can be identified: food, medicine, fuel wood, fodder, and fragrance, oil, dye, and flavor. Similar classifications were found in previous studies. For example, Asif et al. [28] reported five groups of wild plants from tribal communities in the tehsil of Karnah (Jammu and Kashmir), India. Haq et al. [48] classified the wild plants of district Reasi into four plant usage groups. Rivera et al. [80] observed eight major clusters in the mountains of Castilla-La Mancha (Spain). Similarly, multivariate analysis was used by Balemie and Kebebew [81], Leduc et al. [82], Caneva et al. [83], and Haq et al. [84] for quantitative ethnobiological approaches in their studies.

### 3.5. Important Medicinal Plant Species, Their Local Uses, and Trade Status

The local inhabitants collected most plants for self-use (65%) or for income earning (35%). Every single medicinal plant found in the study area is valuable in the local healthcare system, although some have especially high significance value in the traditional “Sowa-Rigpa” healthcare system, e.g., *Aconitum heterophyllum*, *Aconitum violaceum*, *Arnebia guttata*, *Arnebia euchroma*, *Aster flaccidus*, *Bergenia stracheyi*, *Corydalis govaniana*, *Dactylorhiza hatagirea*, *Gentiana algida*, *Hippophae rhamnoides*, *Inula racemosa*, *Jurinea dolomiaea*, *Meconopsis aculeata*, *Picrorhiza kurroa*, *Rhododendron anthopogon*, *Rheum webbianum*, *Rheum spiciforme*, *Saussurea bracteata*, *Saussurea lappa,* and *Vincetaxicum caneces*. *Allium przewalskianum*, *Waldheimia tomentosa,* and *Juniperus indica* (Shukpa) were found to be commonly used by the Ladakhi people as a source of income at the local level (Leh market). Similarly, it was found that plants such as *Allium przewalskianum*, *Amaranthus spinosus*, *Allium humile*, *Plantago depressa,* and *Urtica hyperborea* were mostly used as food, and *Hippophae rhamnoides* was used to make juice in the study area. Plants, in addition to playing a role in treating various ailments [85,86,87], also deliver tangible economic benefits to indigenous communities, as in other regions [88]. The sustainable utilization and management of wild resources can act as a strategy to boost livelihood generation and food security and aid in poverty alleviation. Khan et al. [89], while carrying out a study on the indigenous communities of Ladakh, reported that 14.05 percent of the households surveyed were involved in medicinal plant collection, while only 8.11 percent were involved in marketing. The medicinal plants generated a total income of 274,034.40 annum^−1^ at 1481.27 household^−1^ annum^−1^ and 514.09 man-days annum^−1^ in the sampled population, with an average employment opportunity of 0.35 man-days household^−1^ annum^−1^. In terms of subsistence and income generation, medicinal plants play an important role in aboriginal people’s livelihood support.

## 4. Conclusions

The ethnobotanical results of this study clearly demonstrate that the traditional knowledge of medicinal plants is mainly the asset of elders. A total of 105 plant species belonging to 82 genera and 39 families were documented. The results indicated that the most prominent family is Asteraceae. The plant part most used are leaves. Medicinal uses are the most prevalent, with 70% of use reports, followed by fodder and fuel wood. *Delphinium brunonianum*, *Waldheimia tomentosa*, and *Juniperus indica* play a significant role in the cultural and religious ritual aspects. The local people collect most plants (65%) for self-consumption, while the rest (35%) are sold in markets as a source of income. The comparative analysis with previously published works showed similarities with our data. The results clearly indicate a real risk of progressive loss of traditional knowledge. In this study, some plants were reported for the first time for their ethnomedicinal use. These should be assessed for phytochemical composition and pharmacological activities. Further research on conservation strategies needs to be conducted.

## Figures and Tables

**Table 1:** Demographic details of respondents interviewed in the present study.

Professional Groups Interviewed	Professional Group	Number
	Farmers	82
	Housewives	52
	Herders	24
	Govt. employees	23
	Amchis	21
	Daily wage laborers	21
	Shopkeepers	13
	Hunters	16
	Hotel owners	15
	Museum owners	2
Gender	Male	208
	Female	61
Ethnic group	Balti	91
	Beda	63
	Brokpa	115
Age group	25–45	33
	46–65	128
	65–88	106
Education qualification	Without formal education	174
	5th pass	37
	8th pass	21
	10th pass	15
	12th pass	14
	Graduate and above	8

**Table 2:** List of plant species with their ethnomedicinal usage in the Ladakh trans-Himalayan region, India.

Botanical Name Abbreviation Family Voucher No.	Local Name English Name	Life Form	Parts Used	Preparation and Application	Diseases Cured	Other Economic Applications	Ethnic Groups		
							Balti	Brokpa	Beda
*Amaranthus spinosus* L. (Ama-spi) Amaranthaceae SMH-162	Rgya-sho/ Green amaranth	Herb	Leaves Young twigs	Leaves are ground and paste is used topically. Young twigs are boiled in water and decoction is taken orally.	Leaves are used for wound healing and bone fractures. Young twigs are used to treat pulmonary diseases.	Leaves are used as food.	Y	Y	Y
*Allium humile* Kunth. (All-hum) Amaryllidaceae SMH-164	Sko-tse/Kue Bulb onion	Herb	Bulbs Leaves	Decoction is made from the bulb which is used orally. Fresh leaves are used.	Increases sexual vitality, gynecological disorders, insomnia, appetite, indigestion, and elephantiasis.	Raw bulb is used along with food. Leaves are cooked and used as vegetables.	Y	Y	Y
*Allium przewalskianum* Regel. (All-prz) Amaryllidaceae SMH-129	Hdzim-nag-gam-ri-sGog, Rug pa sa, Lu i ljang pa/ Wild onion	Herb	Bulbs	Bulbs are ground and made into paste and used topically.	Headache and wind diseases.	Bulbs are cooked as food.	Y	Y	N
*Angelica glauca* Edgew. (Ang-gla) Apiaceae SMH-203	Lcha-ba/ Angelica	Herb	Roots	Roots are dried and ground to a powder and taken with lukewarm water and lime.	Restores body strength and kidney heat, balances phlegm diseases, stomachic disorders, dropsy, anemia, and wind diseases.	---------------	Y	Y	N
*Anaphalis triplinervis* (Sims) Sims ex C.B. Clarke. (Ana-tri) Asteraceae SMH-127	Spra-rgod, Spra g -yung, Tayung/ Pearly everlasting	Herb	Leaves	Leaves are ground and made into peanut-like tablets, taken orally with water twice a day.	Epidemic fever, stone, poison, and lymph gland swelling and bleeding.	---------------	Y	Y	Y
*Arctium lappa* L. (Arc-lap) Asteraceae SMH-185	Byi-bzung/ Greater burdock	Herb	Roots Seeds Leaves	Decoction is made from roots and taken orally. Seeds and leaves are ground to a fine powder, which is taken with water for a week. Leaves are ground to a fine powder and taken orally with water.	Roots are believed to have anticancer potential and used against uterus tumors and urinary bladder cysts. Seeds are used to cure burning urination. Leaves are used for nerve disorders and unripe epidemic fever.	---------------	N	Y	N
*Artemisia absinthium* L. (Art-abs) Asteraceae SMH-125	Bur-tse, Khan dkar, Dpa bo nyid/ Wormwood	Herb	Leaves	Tea is obtained by boiling the dry and fresh leaves for more than half an hour and taken orally.	Analgesic during labor pain, leukemia, and sclerosis.	---------------	Y	Y	N
*Artemisia dracunculus* L. (Art-dra) Asteraceae SMH-131	Tse-phat, tshar-bong/ Tarragon	Herb	Leaves	Ground fresh leaves are made into small tablets and taken orally.	Digestive disorders, toothache, pharyngitis, pulmonary diseases, and swelling due to hot disorders.	---------------	Y	Y	N
*Aster flaccidus* Bunge. (Ast-fla) Asteraceae SMH-103	Me-tok-lug-mig, Nia-Mentok/ Alpine aster	Herb	Flowers Leaves	Decoction is made from dry and fresh flowers, taken orally. Paste is made from leaves and applied topically.	Flowers are used against poisoning, epidemic fever, infectious cold and cough, spasms of tendons and ligaments. Leaves are used to treat drying pus and blood of wounds, and blisters.	Flowers are used as ornaments.	Y	Y	N
*Acantholimon lycopodioides* (Girard) Boiss. (Aca-lyc) Plumbaginaceae SMH-128	Long-zay/ Prickly thrift	Herb	Leaves	Dried and fresh leaves are boiled in water to make decoction, which is taken orally twice a day.	Cardiac disorders.	Litter in the form of dry leaves serves as fuel.	Y	Y	Y
*Aconogonum tortuosum* (D. Don) H. Hara. (Aco-tor) Polygonaceae SMH-102	Rnyalo, Chu rtsi, Skyu ru mu ru/ Twisted Knotweed	Herb	Roots	Roots are boiled in water and then taken orally after more than two hours.	Black motion, dysentery, diarrhea, lumbar pain due to delivery.	---------------	Y	Y	N
*Aconitum heterophyllum* Wall. ex Royle. (Aco-het) Ranunculaceae SMH-195	Boona-karpo/ Indian atees	Herb	Roots	Paste is obtained via grinding the roots and applied topically.	Useful in toothache and headache.	---------------	Y	Y	N
*Aconitum richardsonianum* Lauener. (Aco-ric) Ranunculaceae SMH-187	Bong-nag-lo-pohra/ Monkshood	Herb	Roots	Dried roots are ground to a fine powder and taken with lukewarm water orally.	Common cold, pneumonia, laryngitis, croup, and asthma, inflammation, and high blood pressure.	---------------	Y	Y	N
*Aconitum violaceum* Jacquem. ex Stapf. (Aco-vio) Ranunculaceae SMH-163	Boona-nagpo/ Violet monkshood	Herb	Roots	Roots are dried in shade and ground to a powder, taken with lukewarm water.	Cold, cough, asthma, fever, and gastric problems.	---------------	Y	Y	N
*Anemone rivularis* Buch.-Ham. ex DC. (Ane-riv) Ranunculaceae SMH-186	Srub-ka, Sngo srub/ Riverside windflower	Herb	Roots	Dried roots are ground to powder and taken with boiled water orally.	Severe body pain, stomach heat, snake poison, tumors and drying serous fluids.	---------------	Y	Y	Y
*Aquilegia fragrans* Benth. (Aqu-fra) Ranunculaceae SMH-130	Doftakleja/ Fragrant columbine	Herb	Flowers	Fresh flowers are used.	---------------	Flowers are used for ornamental purposes.	Y	Y	Y
*Arnebia euchroma* (Royle) I.M.Johnst. (Arn-euc) Boraginaceae SMH-126	Bri-mok, Demok/ Pink arnebia	Herb	Roots	Roots are shade-dried and decoction is made from ground roots, taken orally for a week.	Blood purifier, cold, cough, lung, and pulmonary problems.	Roots are used as coloring dishes and sweets.	Y	Y	Y
*Arnebia guttata* Bunge (Arn-gut) Boraginaceae SMH-161	Deemok/ Arnebia	Herb	Roots	Roots are shade-dried and decoction is made from ground roots, which is taken orally for three consecutive days.	Reduce cough and cold.	---------------	Y	Y	Y
*Bergenia ciliata* (Haw.) Sternb. (Ber-cil) Saxifragaceae SMH-202	Pasanbheda/ Winter begonia	Herb	Whole plant	Plant is sun-dried, ground to a powder, and taken with water orally.	Renal calculi.	Roots are used for making herbal tea.	Y	Y	Y
*Berberis dictyophylla* Franch. (Ber-dic) Berberidaceae SMH-124	Sker-pa/ Netleaf barberry	Shrub	Bark	Tea is obtained by boiling the bark in water for more than 40 min, and taken orally.	Diabetes, trachoma, and nephritis.	---------------	N	Y	N
*Berberis ulicina* Hook.f. & Thomson. (Ber-uli) Berberidaceae SMH-184	Khizer, Kiraring/ Gorse Barberry	Shrub	Roots Bark	Roots are taken raw, dried and ground to a powder, and used orally with milk.	Both the roots and the bark are used for body strength.	---------------	N	Y	N
*Carum carvi* L. (Car-car) Apiaceae SMH-104	Kos-nyod, Ajaji, Nilisi shi/ Caraway	Herb	Seeds	Seeds are dried and ground to a fine powder; also used in natural form.	---------------	Seeds are used to add flavor to local dishes.	Y	Y	N
*Cortia depressa* (D. Don) C. Norman. (Cor-dep) Apiaceae SMH-158	Bam-po-mo-rig/ Iranian knapweed	Herb	Bulbs Leaves	Bulbs are made into paste and applied topically. Decoction is made from the leaves and taken orally.	Bulbs reduce swelling in limbs. Leaves are used for muscular spasms.	---------------	Y	Y	N
*Carthamus lanatus* L. (Car-lan) Asteraceae SMH-123	Gur-gum/ Safflower	Herb	Flowers	Dried flowers are ground to a powder and taken orally with lukewarm water.	Liver disorders, hepatitis, anemia, fever, purify blood, and bleeding.	---------------	Y	Y	Y
*Cosmos bipinnatus* Cav. (Cos-bip) Asteraceae SMH-121	Pun-da-Re-ka, Krang tha ri/ Garden cosmos	Herb	Roots	Roots are dried and ground, then mixed with water, taken orally.	Stops bleeding and blood fever.	---------------	Y	Y	N
*Cremanthodium ellisii* (Hook.f.) Kitam. (Cre-ell) Asteraceae SMH-188	Ming-chan-nagpo/ Himalayan mini-Sunflower	Herb	Roots Leaves Flowers	Paste is made from the roots and applied topically. Decoction is made from the leaves and used orally. Flowers are hand-rubbed.	Roots are used against inflammation of any body part. Flowers are used to treat larynx disorders. Flowers are used to treat diseases caused by evil spirits.	---------------	Y	Y	N
*Codonopsis ovata* Benth. (Cod-ova) Campanulaceae SMH-181	Klup-dud-rdo-rje/ Kashmir bonnet bellflower	Herb	Leaves Flowers	Poultice is made from leaves and used topically. Flowers are dried and ground to a powder, mixed with water and made into paste, and applied topically.	Leaves are used against arthritis, rheumatism, and elephantiasis. Flowers are used to treat leprosy, nerve disorders, stiffening of ligaments and tendons, planetary diseases, and diseases caused by evil spirits.	---------------	Y	Y	N
*Capparis spinosa* L. (Cap-spi) Capparaceae SMH-160	Kabra/ Flinders rose	Herb	Flowers Fruits	Flowers are dried and ground to a powder.	---------------	Flowers are used as flavoring agent. Fruits are used as food.	Y	Y	N
*Caragana brevifolia* Kom. (Car-bre) Fabaceae SMH-132	Brama/ Caragana	Shrub	Roots Fruits	Paste is made from the roots and applied topically. Fruits are used raw.	Roots are used against muscle and nerve inflammation.	Fruits are used as food.	Y	Y	N
*Caragana tibetica* Kom. (Car-tib) Fabaceae SMH-183	Mdzo-mo-shing/ -----------	Shrub	Roots	Decoction is obtained from the roots and taken orally.	Blood disorders, skin diseases, heart disorders, and eye diseases.	---------------	Y	Y	N
*Caragana versicolor* Benth. (Car-ver) Fabaceae SMH-159	Tama/ ---------	Shrub	Whole plant	---------------	---------------	Litter and the dried parts are used as fuel wood. Leaves are used as fodder.	Y	Y	Y
*Chesneya cuneata* (Benth.) Ali. (Che-cun) Fabaceae SMH-182	Byangbu, Dama/ Wedge-leaf chesneya	Herb	Roots Fruits (beans)	Roots are ground and mixed with lime and water to form tablets, used orally twice a day for a week. Fruits (beans) are cooked.	Roots are used against skin infections.	The beans are eaten as vegetables.	Y	Y	N
*Cicer microphyllum* Benth. (Cic-mic) Fabaceae SMH-196	Sari/ Himalayan *chickpea*	Herb	Whole plant	Plant is dried. Decoction obtained is taken orally.	Exhilarate and purify the blood.	Whole plant is used as feed and fodder. Seeds are used as food (raw or cooked).	Y	Y	Y
*Corydalis govaniana* Wall. (Cor-gov) Papaveraceae SMH-134	Stong-zil/ ----------	Herb	Roots Leaves	Small maize-like pellets are made from the roots by grinding and mixing water. Decoction is made from the leaves and taken orally.	Roots are used as antipyretic and diuretic. Leaves are used against gastric pain, muscular pain, contagious fever, eye diseases, and swellings.	---------------	N	Y	N
*Clematis montana* Buch. -Ham. ex DC. (Cle-mon) Ranunculaceae SMH-133	Dbye-mong-karpo/ Himalayan clematis	Herb	Stem Flowers	Stem and flowers are dried in the sun and ground to a powder, which is used with water in the morning.	Aerial parts (stem and flowers) are used to treat diabetes.	---------------	N	Y	N
*Clematis tibetana* Kuntze. (Cle-tib) Ranunculaceae SMH-122	Dbi-mong, Sbi-cho/ Chinese clematis	Herb	Flowers Stem	Decoction used orally and paste used topically are made from both stem and flowers.	Flowers are used in wind- or cold-related problems, tumors, wounds, and arthritis. Stem is used against pulmonary diseases, digestive heat, burns, and lack of appetite.	---------------	N	Y	N
*Datura stramonium* L. (Dat-str) Solanaceae SMH-205	Esman/ Thorn apple, jimsonweed	Herb	Seeds	Seeds are dried and ground to a powder and taken orally with water. Dried seeds are burned to produce smoke.	Seeds are used against asthma and diarrhea, and as anti-inflammatory medicine. Smoke is used to exorcise evil spirits.	---------------	Y	N	N
*Debregeasia edulis* (Siebold & Zucc.) Wedd. (Deb-edu) Urticaceae SMH-197	Ga—dur/ ----------	Shrub	Fruits	Fresh and dried fruits are used.	---------------	Fruits are used as food.	N	Y	Y
*Delphinium brunonianum* Royle. (Del-bru) Ranunculaceae SMH-105	Bya-rgod-spos/ Musk larkspur	Herb	Stem Leaves	Paste is made from the leaves and used topically. Decoction is obtained from the leaves and taken orally.	Stem is used to treat skin diseases. Leaves are used against evil spirits, poisoning, epidemic fever, itching, cold, cough, and snakebite.	---------------	N	Y	Y
*Delphinium cashmerianum* Royle. (Del-cas) Ranunculaceae SMH-180	Ba-ru-ra, Ladar/ Kashmir larkspur	Herb	Leaves	Fresh leaves are used as such.	Insecticide.	---------------	N	Y	Y
*Dactylorhiza hatagirea* (D.Don) Soó. (Dac-hat) Orchidaceae SMH-201	Ambo-lakpa/ Himalayan marsh orchid	Herb	Roots	Roots are made into decoction and used orally.	Energy boosters; improve health; recommended for weak people.	---------------	Y	Y	N
*Dracocephalum heterophyllum* Benth. (Dra-het) Lamiaceae SMH-179	Jib-rtse-Karpo, Jib pa dkar thog,x Mdzo mo nu nu/ White dragonhead	Herb	Leaves Flowers	Raw leaves are chewed. Boiled in hot water to get vapors.	Leaves are used against toothache and eye diseases. Flowers are used to treat irritation, burning sensation, and pain.	---------------	N	Y	N
*Dracocephalum tanguticum* Maxim. (Dra-tan) Lamiaceae SMH-200	Pri-yang-ku, Klum o gur gum, Btsun mo gur gum/ ----------	Herb	Leaves	Leaves are boiled in water and eaten.	Hepatitis, gastritis, dizziness, arthritis, and stomach ulcer.	---------------	N	Y	N
*Doronicum falconeri* C.B. Clarke ex Hook.f. (Dor-fal) Asteraceae SMH-120	Mentok-serpo/ False leopard’s bane	Herb	Leaves Flowers	Paste is made from both leaves and flowers.	Leaves are used to treat diphtheria and viral and bacterial diseases. Flowers are used to treat swelling and pain due to hypertension.	---------------	N	Y	N
*Echinops cornigerus* DC. (Ech-cor) Asteraceae SMH-135	Aczema/ Blue globe thistle	Herb	Roots Leave	Roots are powdered and mixed with curd and used topically. Leaves are dried and ground to form small tablets and taken with water orally.	Roots are used to treat yellowish coloration of eyes due to jaundice. Leaves are used to treat septic wounds.	---------------	Y	Y	N
*Ephedra gerardiana* Wall. ex Stapf. (Eph-ger) Ephedraceae SMH-119	Tsepat, Chhepat/ Gerard jointfir	Herb	Leaves Stem Flowers	Leaves are made into paste and applied topically. Young stem twigs are cut into the size of a toothbrush. Flowers are made into decoction, used orally.	Leaves are used to reduce fever. Flowers are used to treat hepatic diseases, rheumatism, and bronchial asthma.	Stem is an alternative to toothbrush and paste. Dried stem is also used as fuel.	N	Y	N
*Epilobium latifolium* L. (Epi-lat) Onagraceae SMH-106	Byar-pan-chu-tse/ Dwarf fireweed	Herb	Flowers Leaves	Flowers are made into paste and used topically. Decoction is made from the leaves and used orally.	Flowers are used against dropsy. Leaves are used against urine obstruction, severe pain, fever due to elephantiasis, arthritis, and pimples.	---------------	Y	Y	N
*Fritillaria verticillata* Willd. (Fri-ver) Liliaceae SMH-189	Shri khanta/ ---------	Herb	Bulbs	Bulbs are ground and mixed with honey and taken orally.	Antidote, antitussive, astringent, expectorant, and purgative.	---------------	Y	Y	Y
*Ferula jaeschkeana* Vatke. (Fer-jae) Apiaceae SMH-178	Wild Hing, Tru-nag/ Wild Asafoetida	Herb	Roots	Roots are dried and ground to a powder, mixed with spring water to form a paste, and used topically.	Septic wounds and cuts.	---------------	Y	Y	Y
*Gentiana chirayita* Roxb. ex Flem. (Gen-chi) Gentianaceae SMH-118	Tik-ta/ Indian gentian	Herb	Whole plant	Decoction is made and taken orally.	Febrifuge, carminative, expectorant, laxative, stomachic, anthelmintic, and anti-diarrheal.	---------------	N	Y	N
*Gentiana urnula* Harry Sm. (Gen-urn) Gentianaceae SMH-177	Ganga-chung, Rgyal mtshan ganga chung, Gangs las skyes/ Starfish succulent	Herb	Whole plant	Decoction is made and taken orally.	Prominent laxative, anthelmintic, and anti-diarrheal.	---------------	N	Y	N
*Gentiana algida* Pall. (Gen-alg) Gentianaceae SMH-157	Tiktas, Spang-gyan-karpo, Ta pa ni/ whitish gentian	Herb	Flowers	Flowers are dried, ground, mixed with lime and honey, and taken orally.	To reduce the inflammation of the pharynx, bronchitis, cough, hoarseness of throat, excess sputum, and toxic and epidemic fevers.	---------------	N	Y	N
*Herminium monorchis* (L.) R.Br. (Her-mon) Orchidaceae SMH-198	Bye-lche-lag-pa/ Musk orchid	Herb	Roots	Fresh and dry roots are half ground and boiled in water, and taken orally.	Rejuvenates health, increases sperm count; vital essence; acts as aphrodisiac and improves kidney heat.	---------------	N	Y	N
*Hippophae rhamnoides* L. (Hip-rha) Elaeagnaceae SMH-156	Gla ba tsher ma, Gle tsher, Star ru/ Sea-buckthorn	Herb	Whole plant	Plant is dried and powdered. Decoction is made and taken orally.	Anti-aging, anti-cold, memory sharpener, and energy booster.	Whole plant is used as fuel and fodder.	Y	Y	Y
*Heracleum candicans* Wall. ex DC. (Her-can) Apiaceae SMH-136	Spru-nag/ Cartwheel flower	Herb	Roots	Roots are sun-dried and kept for 1 month, then ground to a powder and mixed with curd to form a paste, applied topically.	Sunburn, skin diseases, and external tumors.	---------------	Y	Y	Y
*Iris lactea* Pall. (Iri-lac) Iridaceae SMH-117	Dres-ma, Mo dres, Modrey/ Milky iris	Herb	Whole plant	Plant is dried and powdered. Decoction is made and taken orally.	Appetite, stomach cramps, small and large intestinal cramps, and food-poisoning diseases.	---------------	Y	Y	N
*Ixeris gracilis* (DC.) Stebbins. (Ixe-gra) Asteraceae SMH-190	Rtga-mkhris/ ---------	Herb	Whole plant	Plant is dried and powdered. Decoction is made and taken orally.	Antihyperglycemic activity, and dysentery.	---------------	N	Y	N
*Inula racemosa* Hook.f. (Inu-rac) Asteraceae SMH-176	Ma-nu, Pushkarmool/ Indian elecampane	Herb	Roots	Dried roots are powdered, mixed with cow milk, and taken orally.	Bronchial asthma, anthelmintic in children, antiseptic, expectorant, and diuretic.	---------------	Y	Y	N
*Jurinea dolomiaea* Boiss. (Jur-dol) Asteraceae SMH-116	Bya-rog-nyungs-ma/ Dhoop	Herb	Leaves Bark	Paste is made from dry leaves and bark and applied topically.	Leaves are used against rheumatic pains. Bark is used as antiseptic.	---------------	N	Y	N
*Juniperus indica* Bertol. (Jun-ind) Cupressaceae SMH-137	Shukpa, Spa ma, Spa ma bdud rtsi zil pa can/ Black juniper	Shrub	Leaves Bark	Raw leaves are used. Fresh and dried barks are smelled.	Bark is used in inflammation and menstrual problems.	Leaves are used as incense by Buddhists, and hence is attributed religious significance. Bark is used in dealing with nightmares in Muslims.	Y	Y	Y
*Juniperus recurva* Buch. -Ham. ex D.Don. (Jun-rec) Cupressaceae SMH-155	Shug-pa-tser-chan/ Drooping juniper	Shrub	Leaves Fruits	Powdered leaves are used with stream water and taken orally. Fruits are taken raw.	Leaves are used to treat kidney problems and muscular spasms. Fruit improves health.	---------------	Y	Y	Y
*Lactuca dissecta* D. Don. (Lac-dis) Asteraceae SMH-107	Khala/ Split-leaf lettuce	Herb	Leaves Stem	Dried leaves and stem are ground, mixed with water to form paste, and applied topically.	Infections of female external genital organs.	---------------	N	Y	Y
*Lonicera spinosa* (Decne.) Jacq. ex Walp. (Lon-spi) Caprifoliaceae SMH-138	Phang-ma/ Spiny Honeysuckle	Herb	Flowers	Flowers are kept in an air-tight jar for more than 40 days for fermentation and then taken orally.	Asthma, headache, and gynecological disorders.	---------------	Y	Y	Y
*Leonurus japonicus* Houtt. Lamiaceae (Leo-jap) SMH-165	Zin-tig/ Oriental motherwort	Herb	Leaves Stem	Leaves are shade-dried and powdered, mixed with water, and taken orally. Decoction is made from the stem and taken orally.	Leaves regulate menstrual disturbance, dysmenorrhea, amenorrhea, blood stasis, and postpartum hemorrhage. Stem is used as diuretic.	---------------	N	Y	N
*Lagotis cashmeriana* Rupr. (Lag-cas) Plantaginaceae SMH-175	Hong-len/ Kashmir *lagotis*	Herb	Roots	Roots are shade-dried and boiled in water and taken orally.	Reduce inflammation, vital fever, liver fever, lungs fever, intestinal fever, poisonous, painful cramps, muscular spasms, and child dysentery.	---------------	N	Y	N
*Myricaria squamosa* Desv. (Myr-squ) Tamaricaceae SMH-174	Om-bu/ Scaly false tamarisk	Shrub	Stem	Dried stem is ground and decoction is made; taken orally.	Food poisoning.	---------------	Y	Y	N
*Myricaria elegans* Royle. (Myr-ele) Tamaricaceae SMH-115	Hom-bu/ Elegant false tamarisk	Shrub	Stem	Stem is ground and boiled in water for 20 min and then eaten.	Fever of lungs, diarrhea, arthritis, uterine bleeding, stomach pain, and headache.	---------------	Y	Y	N
*Meconopsis aculeata* Royle. (Mec-acu) Papaveraceae SMH-154	Ud-pal-sngon-po/ Poppy	Herb	Leaves	Fresh leaves are powdered, mixed with water to make a paste, and applied topically.	Headache.	---------------	N	Y	Y
*Morina longifolia* Wall. ex DC. (Mor-lon) Caprifoliaceae SMH-191	Spyang-tsher, Bya tra pad, Chang tsher karpo/ Himalayan whorl flower	Herb	Roots Young—twigs	Decoction is made from the roots and young twigs and taken orally.	Roots are used against phlegm disease, cancer, and swelling. Young twigs are used for indigestion.	---------------	Y	Y	N
*Nepeta floccosa* Benth. (Nep-flo) Lamiaceae SMH-153	Shangukuram/ Woolly catmint	Herb	Leaves	Dried leaves are ground to a powder.	---------------	Leaves add flavor in local dishes.	Y	Y	Y
*Oxytropis microphylla* (Pall.) DC. (Oxy-mic) Fabaceae SMH-139	Tag-sha nagpo, Sngo stag sha, Pa nig ho ra/ Small leaved locoweed	Herb	Whole plant	Fresh plant is used.	---------------	The whole plant bears a strong fragrance and hence is kept in homes.	Y	Y	N
*Onosma hookeri* C.B. Clarke. (Ono-hoo) Boraginaceae SMH-108	Bri-mog, xi hua dian zi cao/ ----------	Herb	Roots Bark Leaves	Decoction is made from the roots, bark, and leaves and used orally.	Roots are used against asthma, cough, and fever. Bark is used against gonorrhea and leprosy. Leaves are used against phthisis.	---------------	Y	Y	Y
*Picris hieracioides* Sibth. & Sm. (Pic-hie) Asteraceae SMH-152	Rgya-mkhur, Aam dkar, Gyakhur/ Hawkweed oxtongue	Herb	Whole plant	Plant is dried and powdered, mixed with water, and taken orally.	Gastro-intestinal disorder, blood disorder, bile disorder, chronic fever, and contagious fever.	---------------	N	Y	Y
*Pedicularis cheilanthifolia* Schrenk. (Ped-che) Orobanchaceae SMH-192	Lug-ru-smog-po, sui mi jue ye/ White Lousewort	Herb	Stem	Stem is dried and ground to a fine powder and taken with water orally.	Stomach ache.	---------------	Y	Y	Y
*Pedicularis longiflora* Rudolph. (Ped-lon) Orobanchaceae SMH-166	Lug-ru-ser-po/ Long tube lousewort	Herb	Flowers	Decoction is obtained from dried flowers and used orally.	Diuretic, liver, and gallbladder disorders, excessive seminal discharge, and edema.	---------------	Y	Y	Y
*Piper longum* L. (Pip-lon) Piperaceae SMH-173	Pi-pi-ling, Byi ril ma, Yul dbus/ Long pepper	Herb	Roots Fruits	Roots and fruits are dried and boiled in water for some time and then taken orally.	Prevention and treatment of prostate cancer.	---------------	Y	Y	Y
*Plantago depressa* Willd. (Pla-dep) Plantaginaceae SMH-140	Tha-ram/ Blond psyllium	Herb	Leaves Stem Flowers	Leaves are dried, powdered, and taken with lukewarm water orally. Fresh stem is ground, made into a paste, and applied topically. Decoction is obtained from the flowers and used orally.	Leaves are used against dysentery. Stem is used against burn wounds. Flowers are used against bleeding and inflammation.	---------------	N	Y	Y
*Polygonum aviculare* L. (Pol-avi) Polygonaceae SMH-151	Byi-na-sa/ Knotgrass	Herb	Whole plant	Decoction is obtained by grinding and boiling in water for more than 3 h and taken orally.	Obstruction of urine, burning sensation during urination, jaundice, and dermatological diseases with itching.	---------------	Y	Y	Y
*Potentilla anserina* L. (Pot-ans) Rosaceae SMH-144	Gro-lo sa-hdzin, Gro ma, Dolo sazin/ Silverweed	Herb	Leaves	Leaves are dried in homes, powdered, and used orally with water.	Diarrhea/dysentery, and health tonic.	---------------	Y	Y	Y
*Picrorhiza kurroa* Royle. (Pic-kur) Scrophulariaceae SMH-199	Hong-len, Kutki/ Katuk	Herb	Roots Flowers	Roots are dried in shade, powdered, mixed with water, and taken orally. Dried flowers are hand-rubbed into lukewarm water and used orally.	Roots are used to treat asthmatic disorders and fever. Flowers are used in blood purification.	---------------	N	Y	N
*Physochlaina praealta* (Decne.) Miers. (Phy-pra) Solanaceae SMH-109	Lantang, Thang-phron-nagpo/ Praealtus,-a,-um	Herb	Seeds Leaves	Seeds are ground to a fine powder and used orally with lukewarm water. Fresh leaves are ground to obtain the juice, which is used as eye drops.	Seeds are used as vermifuge. Leaves are used against eye diseases.	---------------	Y	Y	N
*Rhodiola imbricata* Edgew. (Rho-imb) Crassulaceae SMH-143	Shro lo, Cho-ngo-ngo/ Greek rhodon	Herb	Roots	Roots are boiled in water, mixed with lime, and taken orally.	Lung problems, cold, cough, fever, loss of energy, and pulmonary complaints.	---------------	Y	Y	Y
*Rhodiola sacra* Prain ex Raym. Hamet S.H. Fu. (Rho-sac) Crassulaceae SMH-193	Srolo-marpo/ Arctic root	Herb	Roots Leaves	Roots are dried and ground and taken orally with water. Fresh young leaves are used.	Roots are used as tonic and to restore memory.	Leaves are cooked as vegetables.	Y	Y	Y
*Rhododendron anthopogon* D. Don. (Rho-ant) Ericaceae SMH-111	Ba-lu/da-lis/ Brass	Shrub	Leaves Flowers	Leaves and flowers are dried, hand-rubbed, kept in hot water for some time, and taken orally.	Leaves are used to treat high-altitude diseases, chest pain, weakness, obstruction of vocal cords, and chronic bronchitis. Flowers are used against stiffness of limbs, and phlegm disorders.	---------------	N	Y	Y
*Ribes orientale* Desf. (Rib-ori) Grossulariaceae SMH-141	Askuta, Se-rgod/ Oriental gooseberry	Shrub	Fruits	Raw fruits are used.	Swollen limbs, food poisoning, hepatitis, fever of the gallbladder, and epidemic fever.	---------------	N	Y	N
*Rheum webbianum* Royle. (Rhe-web) Polygonaceae SMH-150	Chu-rtsa/ Indian rhubarb	Herb	Root Stem Leaves	Decoction is made from fresh roots and taken orally. Fresh stem is made into a paste and applied topically. Raw leaves are taken orally.	Roots are used against indigestion and abdominal diseases. Stem is used to treat boils and wounds. Leaves are used against gastritis and as laxative and astringent.	---------------	Y	Y	N
*Ranunculus adoxifolius* (Ran-ado) Hand. -Mazz. Ranunculaceae SMH-110	Ga-tsah-am-lche-tsah/ Buttercup	Herb	Leaves Flowers	Decoction is made from leaves and taken orally. Fresh flowers are made into a paste, applied topically.	Leaves are used to normalize the digestive heat. Flowers are used against old wounds and dropsy.	---------------	N	Y	Y
*Rosa sericea* Wall. ex Lindl. (Ros-ser) Rosaceae SMH-149	Shayh/ Silky rose	Shrub	Whole plant	Litter and dried plant/ fresh plant is used.	---------------	Whole plant is an ornamental hedge. Plant is also used as fuel and fodder.	Y	Y	Y
*Rosa webbiana* Wall. ex Royle. (Ros- web) Rosaceae SMH-204	Seba, Sia Webb’s rose	Shrub	Flowers	Fresh flowers are used.	---------------	Flowers are used as an ornaments.	Y	Y	Y
*Rubia cordifolia* L. (Rub-cor) Rubiaceae SMH-168	Btsod/ Common madder	Herb	Stem	Decoction taken orally is made from dried stem.	Blood disorders, blood fever, spreading fever, lung fever, kidney fever, and intestine fever.	---------------	N	Y	Y
*Rheum spiciforme* Royle. (Rhe-spi) Polygonaceae SMH-167	Lachhu/ Spiked rhubarb	Herb	Roots	Roots are boiled in water and then used orally.	Rheumatism.	---------------	Y	Y	Y
*Salix pycnostachya* Andersson. (Sal-pyc) Salicaceae SMH-206	Malchang/ Willow	Tree	Branches Stem Leaves	Fresh branches are used. Dried stem is used. Fresh leaves are used.	---------------	Muslims use branches as pillows for corpses in the graves. Stem is used as fuel wood. Leaves are used as fodder.	Y	N	N
*Stipa orientalis* Trin. ex Ledeb. (Sti-ori) Poaceae Malchang	Pilli, Makpen/ Feather grass	Herb	Whole plant	Both fresh and dried plants are used.	---------------	Whole plant is used as fuel and fodder.	Y	Y	N
*Stachys tibetica* Vatke. (Sta-tib) Lamiaceae SMH-145	Yakzas/ Tibetan woundwort	Herb	Whole plant	Dried plant is ground to a fine powder, mixed with curd, and taken orally.	Mental disorders and phobias.	Whole plant is used as fuel and fodder.	Y	Y	N
*Sedum ewersii* Ledeb. (Sed-ewe) Crassulaceae SMH-169	Srolo-karpo/ Pink sedum	Herb	Whole plant	Fresh and dried plants are used.	Plant is given in bulk to the cattle to increase milk production.	---------------	Y	Y	Y
*Saussurea bracteata* Decne. (Sau-bra) Asteraceae SMH-148	Spang-rtsa/ Narrow-leaved saw-wort	Herb	Flowers	Paste is made from flowers and applied topically.	Boils.	Flowers are used as ornaments.	Y	Y	Y
*Saussurea lappa* (Decne.) Sch.Bip. (Sau-lap) Asteraceae SMH-112	Kuth, Ru-ta/ Kust	Herb	Roots	Roots are powdered and taken with water orally.	Asthma and fever.	---------------	Y	Y	Y
*Saussurea roylei* C.B. Clarke. (Sau-roy) Asteraceae SMH-142	Kon-pa-gab-skyes-che-ba/ Royle’s saw-wort	Herb	Leaves	Fresh flowers are made into a decoction and taken orally.	Chronic and fresh wounds, bleeding due to nerve disorders.	---------------	Y	Y	Y
*Tylophora fasciculata* Buch. -Ham. ex Wight. (Tyl-fas) Apocynaceae SMH-194	Go-snyod/ Country ipikakyun	Herb	Fruits	Raw fruits are eaten.	Dysentery and hot disorder of gallbladder.	---------------	N	Y	N
*Tanacetum dolichophyllum* (Kitam.) Kitam. (Tan-dol) Asteraceae SMH-113	Mkhan-chung-ser-mgo/ Garden tansy	Herb	Leaves Flowers	Fresh leaves are ground, and a little juice is obtained and taken orally. Flowers are boiled in water for 5 min and then taken orally with water.	Leaves are used against nose bleeding, swelling, and inflammation of the limbs, cancers, wounds, and lung disorders. Flowers are used against renal diseases.	---------------	N	Y	Y
*Tanacetum gracile* Hook.f. & Thomson. (Tan-gra) Asteraceae SMH-146	Khamchu/ Daisy	Herb	Leaves Flowers	Leaves and flowers are shade-dried. Flowers are shade-dried and covered with a fine cotton cloth.	---------------	Leaves are a good source of oil. Flowers are fragrant and a good source of oil.	N	Y	Y
*Thlaspi arvense* L. (Thl-arv) Brassicaceae SMH-170	Bre-ga/ Field pennycress	Herb	Leaves	Leaves are dried and powdered; decoction is made and taken orally.	Pulmonary diseases, kidney diseases, and white discharges.	---------------	Y	Y	Y
*Trigonella emodi* Benth. (Tri-emo) Fabaceae SMH-147	Hbu-su-hang/ Himalayan fenugreek	Herb	Seeds Leaves	Seeds are dried, ground, mixed with turmeric to form a paste, and applied topically. Leaves are dried and powdered; decoction is made and taken orally.	Seeds are used against sores and bone fractures. Leaves are used to treat kidney disorders.	---------------	Y	Y	N
*Urtica hyperborea* Jacq. ex Wedd. (Urt-hyp) Urticaceae SMH-171	Rza-sot/ Northern nettle	Herb	Leaves	Leaves are dried, powdered, and taken orally with lukewarm water.	Promotes digestive and physical heat, and wind diseases associated with chronic fever.	Leaves are used as food (soup).	N	Y	Y
*Waldheimia tomentosa* (Decne.) Regel. (Wal-tom) Asteraceae SMH-114	Palu/ Yellow berried nightshade	Herb	Whole plant	Decoction is made and used orally. Fresh and dried plants are used.	Acidity, headache, and arthritis.	Whole plant is considered holy in the Buddhist faith and used for making incense (dhoop), which is used in houses and religious places. Plant is also used in the bathing ceremony of deceased persons and newborn babies.	Y	Y	Y
